# Multiparameter diagnostic model based on ^18^F-FDG PET and clinical characteristics can differentiate thymic epithelial tumors from thymic lymphomas

**DOI:** 10.1186/s12885-022-09988-1

**Published:** 2022-08-16

**Authors:** Guanyun Wang, Lei Du, Xia Lu, Jiajin Liu, Mingyu Zhang, Yue Pan, Xiaolin Meng, Xiaodan Xu, Zhiwei Guan, Jigang Yang

**Affiliations:** 1 Nuclear Medicine Department, Beijing Friendship Hospital, Capital Medical University, 95 Yong’an Road, Xicheng District, Beijing, 100050 China; 2 Department of Nuclear Medicine, The First Medical Center, Chinese PLA General Hospital, No. 28 Fuxing Road, Haidian District, Beijing, 100853 China

**Keywords:** PET, Thymic epithelial tumors, Thymic lymphomas, Multiparameter, Metabolic parameters, Differential diagnosis

## Abstract

**Objective:**

To evaluate the diagnostic performance of combined multiparametric ^18^F-fluorodeoxyglucose positron emission tomography (^18^FDG PET) with clinical characteristics in differentiating thymic epithelial tumors (TETs) from thymic lymphomas.

**Patients and methods:**

A total of 173 patients with 80 TETs and 93 thymic lymphomas who underwent ^18^F-FDG PET/CT before treatment were enrolled in this retrospective study. All patients were confirmed by pathology, and baseline characteristics and clinical data were also collected. The semi-parameters of ^18^F-FDG PET/CT, including lesion size, SUVmax (maximum standard uptake value), SUVmean (mean standard uptake value), TLG (total lesion glycolysis), MTV (metabolic tumor volume) and SUVR (tumor-to-normal liver standard uptake value ratio) were evaluated. The differential diagnostic efficacy was evaluated using the receiver operating characteristic (ROC) curve. Integrated discriminatory improvement (IDI) and net reclassification improvement (NRI), and Delong test were used to evaluate the improvement in diagnostic efficacy. The clinical efficacy was evaluated by decision curve analysis (DCA).

**Results:**

Age, clinical symptoms, and metabolic parameters differed significantly between patients with TETs and thymic lymphomas. The ROC curve analysis of SUVR showed the highest differentiating diagnostic value (sensitivity = 0.763; specificity = 0.888; area under the curve [AUC] = 0.881). The combined diagnostics model of age, clinical symptoms and SUVR resulted in the highest AUC of 0.964 (sensitivity = 0.882, specificity = 0.963). Compared with SUVR, the diagnostic efficiency of the model was improved significantly. The DCA also confirmed the clinical efficacy of the model.

**Conclusions:**

The multiparameter diagnosis model based on ^18^F-FDG PET and clinical characteristics had excellent value in the differential diagnosis of TETs and thymic lymphomas.

**Supplementary Information:**

The online version contains supplementary material available at 10.1186/s12885-022-09988-1.

## Introduction

Thymic tumors were the most common primary tumor of anterior mediastinum, mainly including thymic epithelial tumors (TETs), thymic lymphomas and germ cell tumors (GCTs) [1, 2]. Thymic epithelial tumors mainly include thymomas, thymic carcinomas, and thymic neuroendocrine tumors (NETs), while major histologic subtypes of thymic lymphomas are primary mediastinal large B-cell lymphoma (PMBCL), nodular sclerosis Hodgkin lymphoma (NSHL), and T-cell lymphoblastic lymphoma (T-LBL) [3, 4]. TETs and thymic lymphomas can account for more than 50% of all anterior mediastinal tumors, and they are the two most common thymic tumors [5–7]. For different types of anterior mediastinal tumors, the treatment options are also totally different. Most TETs are usually treated with surgery, while surgery should be avoided for malignant lymphoma and advanced thymomas/thymic carcinomas, and the systemic treatment for advanced thymomas/thymic carcinomas and lymphoma are also different [8–10]. Therefore, accurate differential diagnosis of anterior mediastinal solid tumors is important for the choice of the treatment strategy.

Although computed tomography (CT) and magnetic resonance imaging (MRI) are often used to evaluate thymic tumors [8, 11], there are still some limitations in distinguishing histological subtypes and definite staging [12]. ^18^F-fluorodeoxyglucose (^18^F-FDG) positron emission tomography/CT (PET/CT) has shown important roles in the management of thymic tumors, including differential diagnosis, predicting stage and classification, evaluation of treatment response and prognosis [10, 13–16]. Analysis based on PET image can provide special information of thymic tumors through qualitative (visual) and semi-quantitative methods (such as calculating maximum standard uptake value [SUVmax], mean standard uptake value [SUVmean], metabolic tumor volume [MTV] and total lesion glycolysis [TLG]) [17–19]. Furthermore, PET/CT can provide more imaging evidence for the evaluation of thymic neoplasms through the metabolic information of PET and the morphological information of CT.

Although several studies have described the ^18^F-FDG PET or PET/CT values in the diagnosis of mediastinal tumors, to our knowledge, most studies have analyzed some types of these tumors [17, 20–22],and there were few systematic descriptions of thymic tumors and comparisons between TETs and thymic lymphomas [23]. Most studies only rely on PET metabolic parameters for analysis, ignoring the differences in patients’ baseline characteristics and clinical symptoms. Based on this, we hope that the combination of ^18^F-FDG PET metabolic parameters and clinical information can play more important role in the differential diagnosis of TETs and thymic lymphomas. Thus, first, we aimed to reveal the difference of ^18^F-FDG PET metabolic parameters between TETS and thymic lymphoma and the effectiveness of PET metabolic parameters in differential diagnosis. Second, we constructed the differential diagnosis model between TETs and thymic lymphomas by combining baseline, clinical data and metabolic parameters, and evaluated the performance of these models.

## Materials and methods

### Patients

This retrospective cohort study, conducted at two institutions (Beijing Friendship Hospital of Capital Medical University and Chinese PLA General Hospital), was approved by the local ethical review boards (The Institutional Review Board of Beijing Friendship Hospital of Capital Medical University and The Institutional Ethics Committee of the General Hospital of the People’s Liberation Army). All patients were informed and signed before ^18^F-FDG PET/CT. It was performed in accordance with the Declaration of Helsinki.

**Fig. 1:**
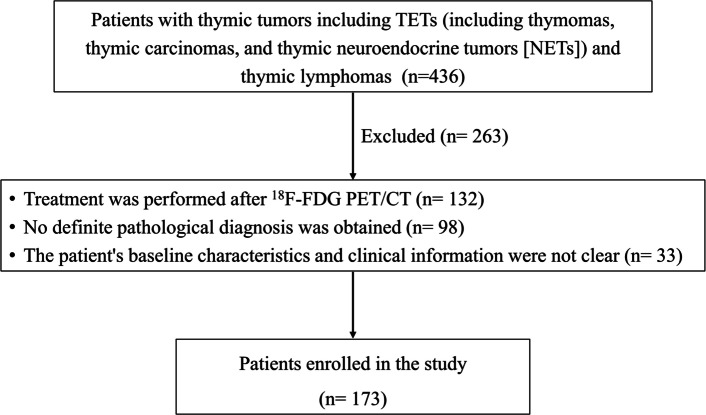
Patient in- and exclusion flow diagram

Between January 2013 and May 2022, a total of 436 patients with thymic tumors including TETs (including thymomas, thymic carcinomas, and thymic NETs) and thymic lymphomas were retrospectively recruited. The inclusion criteria were as follows:(1) ^18^F-FDG PET/CT was performed before treatment; (2) The diagnosis of primary thymic tumors was confirmed by fine-needle biopsy or complete surgical resection pathology; (3) The patient’s baseline characteristics (including age, sex, and body mass index [BMI]) and clinical data (including B symptom, myasthenia gravis, chest pain and respiratory symptoms) were complete. Finally, A total of 173 patients were included in this study (Fig. 1). All thymic tumors were classified based on the fifth edition of the World Health Organization (WHO) classification of Thoracic Tumors [2]. TETs and thymic lymphomas were staged according to Masaoka stage [24] and Ann Arbor stage [25], respectively. We reviewed medical records, PET data, and pathology data of all patients.

### PET/CT protocol

All patients were scanned with ^18^F-FDG PET/CT (Discovery VCT, GE Healthcare, USA/Biograph 64, Siemens Healthineers, Germany/Biography mCT, Siemens Healthineers, Germany). Patients fasted for at least 6 h. The blood glucose level measured before injection was lower than 11.1 mmol/l. The patients rested for 20–30 min in a quiet waiting room before intravenous administration of ^18^F-FDG. An activity of 3.5–4.5 MBq/kg of ^18^F-FDG was administered intravenously. After initial low-dose CT (The parameters of CT included voltage = 120-140 kV, current = 100-200mAs, rotation = 0.8, layer thickness = 3-5 mm, pitch = 0.9–1), a standard PET image [The parameters of PET included 3-dimensional mode, 2–2.5 min/bed (30% overlap), 4–5 beds/person, three iterations, 21 subsets, Gaussian filter half-height width = 4.0 mm] was acquired 45–60 min after injection in free-breathing mode from the skull basis to the upper femur [26]. The images were reconstructed with CT attenuation correction (AC) by using the ordered subset expectation maximization algorithm.

### Image analysis

A centralized reading of the cases was performed by two experienced physicians of nuclear medicine (WGY and DL, with 5 and 10 years of working experience respectively) who blinded to the clinical information of the patients, in consensus on a commercially available workstation (Advantage Workstation 4.6, GE HealthCare). Areas with abnormal uptake of ^18^F-FDG on PET and/or abnormal density on CT were defined as lesions. A two-dimensional region of interest (ROI) was delineated manually according to the boundary of the tumor lesion on each layer of transaxial CT images to form a three-dimensional volume of interest (VOI). There was some essential differences between the two PET/CT systems in machine design and scintillation detection, which may confuse the SUVmax measurement results at least to some extent [27]. To solve this issue, we retrospectively calculated SUVmean of liver parenchyma in the 173 patients of whom the original PET/CT images were available (GE Discovery VCT, *n* = 71; Siemens Biograph 64, *n* = 82; Siemens Biograph mCT, *n* = 20) [28]. To measure normal liver parenchyma activity, 3 non-overlapping spherical 1-cm^3^-sized VOIs were drawn in the normal liver on the axial PET images. There were no significant differences in terms of SUVmean-liver among the 3 PET/CT scanners (GE Discovery VCT, 1.78 ± 0.47 vs. Siemens Biograph 64, 1.77 ± 0.37 vs. Biograph mCT 1.92 ± 0.48, respectively; *F* = 0.957, *P* = 0.386, Variance analysis).

The parameters of PET/CT included lesion size (diameters, mm), SUVmax, SUVmean, MTV (metabolic tumor volume), TLG (total lesion glycolysis, SUVmean×MTV), SUVR (tumor-to-normal liver standard uptake value ratio, SUVmax of the tumor /SUVmean of the normal liver parenchyma). MTV were measured from attenuation-corrected ^18^F-FDG-PET images by two nuclear medicine physicians (WGY and DL) respectively in making these measurements. Because of its high inter-observer reproducibility, the threshold method based on 41% of the SUVmax [29].

### Statistical analysis

Qualitative data are described as number of cases and percentage [n (%)] for categorical variables and quantitative data are described as mean ± SD (standard deviation) for continuous variables. The Mann-Whitney test or student *t* test were used to compare ^18^F-FDG PET/CT parameters between TETs and thymic lymphomas. The area under the receiver operating characteristic (ROC) curve was calculated to assess the predictive value of PET parameters. We calculated sensitivity, specificity, positive predictive value (PPV) and negative predictive value (NPV), respectively. The multivariate logistic regression analysis was used to construct diagnostic models for distinguishing TETs from thymus lymphomas. The bootstrap test, integrated discriminatory improvement (IDI) and net reclassification improvement (NRI) were calculated for comparison of diagnostic models and metabolic parameters with the highest area under the curve (AUC). The IDI and NRI were performed with the *PredictABEL* package, and the bootstrap test was performed with the *pROC* package. To estimate the clinical utility and accuracy of the diagnostic models, decision curve analyses were performed by calculating the net benefits for a range of threshold probabilities in metabolic parameters with the highest AUC and the diagnostic models [30]. DCA was performed with the *rmda* package. The statistical analysis was performed by using commercially available software (IBM SPSS Statistics 24, IBM, Armonk, NY; and R software program, version 4.0.2, Bell Laboratories, USA). All statistical tests were two-tiled and the significance level was set at *P* = 0.05.

## Results

### Clinical characteristics

Table 1 showed the baseline characteristics of 80 TETs and 93 thymic lymphomas patients. The results showed that age (50.83 ± 14.8 vs. 30.3 ± 14.6, *P*<0.001), B symptom (5%, 4 of 80 patients vs. 40%, 37 of 93 patients, *P*<0.001), myasthenia gravis (8%, 6 of 80 patients vs 0%, 0 of 93 patients, *P* = 0.009), chest pain (34%, 27 of 80 patients vs. 15%, 14 of 93 patients, *P* = 0.004) and respiratory symptoms (16%, 13 of 80 patients vs. 36%, 33 of 93 patients, *P* = 0.006) were statistically different between the TETs and thymic lymphomas groups. Compared with TETs, the pathological acquisition methods of patients with thymic lymphoma all based on percutaneous biopsy (65%, 52 of 80 TETs patients vs. 100%, 93 of 93 thymic lymphomas patients, *P*<0.001).

**Table 1:** Baseline and clinical characteristics between thymic epithelial tumors and thymic lymphomas

	Thymic Epithelial Tumors (*n* = 80)		Thymic Lymphoma (*n* = 93)		*P*-value
**Age**	50.8 ± 14.8		30.3 ± 14.6		<0.001*
**Sex**	49:31		52:41		0.537
**(Male:Female, n, %)**	(61%: 39%)		(56%: 44%)		
**Initial major symptoms (n, %)**					
B symptom	4 (5%)		37 (40%)		<0.001
Myasthenia gravis	6 (8%)		0 (0%)		0.009
Chest pain	27 (34%)		14 (15%)		0.004
Respiratory symptoms	13 (16%)		33 (36%)		0.006
**Pathologic procedure (n, %)**					<0.001
Surgery	28 (35%)		0 (0%)		
Percutaneous biopsy	52 (65%)		81 (100%)		
**Histologic type (n, %)**					
	Low-risk thymoma	11 (14%)	Large B-cell lymphoma	37 (40%)	
	Type A thymoma	1 (1.3%)	Hodgkin lymphoma	31 (38%)	
	Type AB thymoma	5 (6.3%)	T lymphoblastic lymphoma	23 (25%)	
	Type B1 thymoma	4 (5%)	MALT lymphoma	1 (1%)	
	Micronodular thymoma	1 (6.3%)	ALCL lymphoma (ALK+)	1 (1%)	
	High-risk thymoma (B2, B3)	17 (21%)			
	Type B2 thymoma	5 (6%)			
	Type B3 thymoma	12 (15%)			
	Thymic carcinoma	44 (55%)			
	Squamous cell carcinoma	32 (40%)			
	Adenocarcinoma	4 (5%)			
	Adenosquamous carcinoma	2 (3%)			
	Sarcomatoid carcinoma	5 (6%)			
	Mucoepidermoid carcinoma	1 (1%)			
	Thymic neuroendocrine tumors	8 (10%)			
**Stage (n, %)**	**Masaoka Stage**		**Ann Arbor stage**		
	I	19 (24%)	I	2 (3%)	
	II	8 (10%)	II	29 (31%)	
	III	10 (13%)	III	11 (14%)	
	IV	43 (53%)	IV	51 (63%)	

*Student *t* test

*MALT lymphoma* Extranodal marginal zone lymphoma of mucosa associated lymphoid tissue, *ALCL lymphoma* Anaplastic large cell lymphoma, *ALK* Anaplastic lymphoma kinase

The pathologic results demonstrated that 11 patients had low-risk thymomas (types A [*n* = 1], AB [*n* = 5] and B1 [*n* = 4], and micronodular type [*n* = 1]); 17 high-risk thymomas (types B2 [*n* = 5] and B3 [*n* = 12]); 44 thymic carcinomas (squamous cell carcinoma [SCC, *n* = 32], adenocarcinoma [*n* = 4], adenosquamous carcinoma [*n* = 2], sarcomatoid carcinoma [*n* = 5], mucoepidermoid carcinoma [*n* = 1]), and 8 thymic neuroendocrine tumors; 37 large B-cell lymphomas, 31 Hodgkin lymphomas, 23 T lymphoblastic lymphomas, 1 MALT (Extranodal marginal zone lymphoma of mucosa associated lymphoid tissue) lymphoma, and 1 ALC (Anaplastic large cell) lymphoma (ALK+) (Table 1). The Masaoka stage was I in 19 (24%), II in 8 (10%), III in 10 (12%), and IV in 43 TETs patients (54%), and the Ann arbor stage was I in 2 (2%), II in 29 (31%), III in 11 (12%), and IV in 51 thymic lymphomas patients (55%).

### Comparison of PET/CT parameters between TETS and thymic lymphomas

**Fig. 2:**
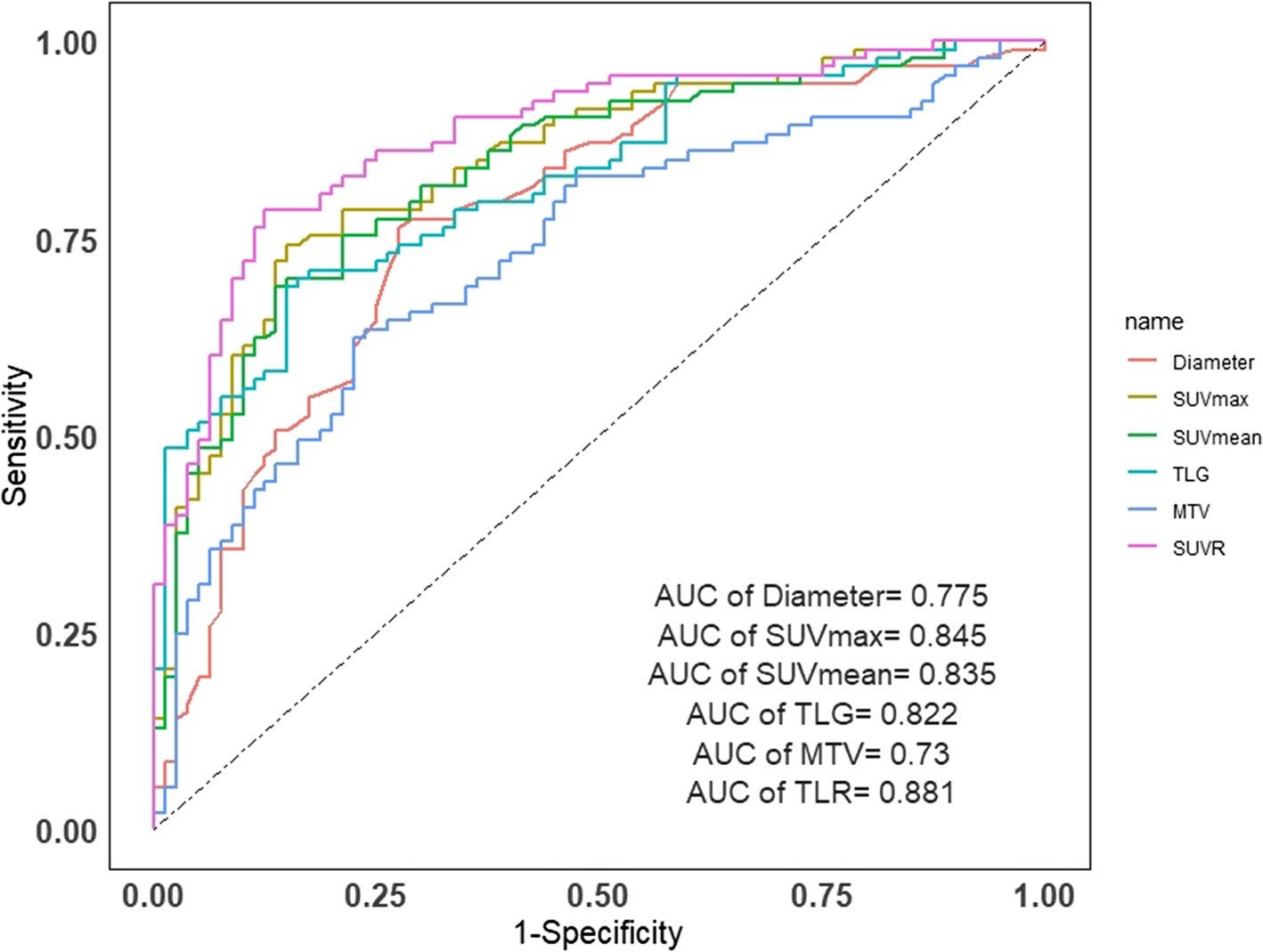
The ROC curves of ^18^F-FDG PET/CT parameters. The areas under the ROC curves for the ability to differentiate TETs from thymic lymphomas for SUVR was 0.881

Comparisons of PET/CT parameters between TETs and thymic lymphomas were shown in Table 2 and Fig. 2. Overall, there were significant differences between TETs and thymic lymphomas groups in diameter (64.1 ± 32.0 vs. 99.9 ± 7.3, *P*<0.001), SUVmax (7.2 ± 4.3 vs. 15.5 ± 7.6, *P*<0.001), SUVmean (4.1 ± 2.5 vs. 8.8 ± 4.6, *P*<0.001), TLG (364.8 ± 482.5 vs. 1927.7 ± 2030.1, *P*<0.001), MTV (92.3 ± 124.1 vs. 228.4 ± 258.4, *P*<0.001) and SUVR (3.7 ± 2.4 vs. 10.5 ± 6.3, *P*<0.001). The ^18^F-FDG PET/CT parameters of different thymic tumors was shown in Table S1.

**Table 2:** The value of ^18^F-FDG PET/CT parameters between thymic epithelial tumors and thymic lymphomas

	TETs	Thymic Lymphomas	*P*
**Lesion size (mm)**	64.1 ± 32.0	99.9 ± 7.3	<0.001*
**SUVmax**	7.2 ± 4.3	15.5 ± 7.6	<0.001
**SUVmean**	4.1 ± 2.5	8.8 ± 4.6	<0.001
**TLG**	364.8 ± 482.5	1927.7 ± 2030.1	<0.001
**MTV**	92.3 ± 124.1	228.4 ± 258.4	<0.001
**SUVR**	3.7 ± 2.4	10.5 ± 6.3	<0.001

*Student *t* test

*SUVmax* Max standard uptake value, *SUVmean* Mean standard uptake value, *MTV* Metabolic tumor volume, *TLG* Total lesion glycolysis, *SUVR* Standard uptake value ratio

### The differentiating efficacy of PET metabolic parameters and diagnostic models in TETs and thymic lymphomas

The ROC analysis indicated that the SUVR showed the highest differentiating diagnostic value in PET/CT parameters with a cut-off value of 6.2 (sensitivity = 0.763, specificity = 0.888, PPV = 0.868, NPV = 0.805, AUC = 0.881). We constructed three different diagnostic models based on multivariate logistic regression analysis, including model 1: age + SUVR, model 2: symptoms+SUVR and model 3: age + symptoms+SUVR. The model 3 with SUVR, age and symptoms (including B symptom, myasthenia gravis, chest pain and respiratory symptoms) resulted in a highest AUC of 0.964 (95% CI: 0.939–0.989), sensitivity = 0.882, specificity = 0.963, PPV = 0.965, NPV = 0.875. The model 3 is shown below.

$$\mathrm{y}=\frac{1}{1+{\mathrm{e}}^{\hbox{-} \left(0.60\times SUVR\hbox{-} 0.08\times \mathrm{Age}+1.70\times \mathrm{B}\kern0.5em symptom\hbox{-} 19.61\times Myasthenia\kern0.5em gravis\hbox{-} 2.67\times Chest\kern0.5em pain+1.18\times Respiratory\kern0.5em symptom s\hbox{-} 0.39\right)}}$$

**Fig. 3:**
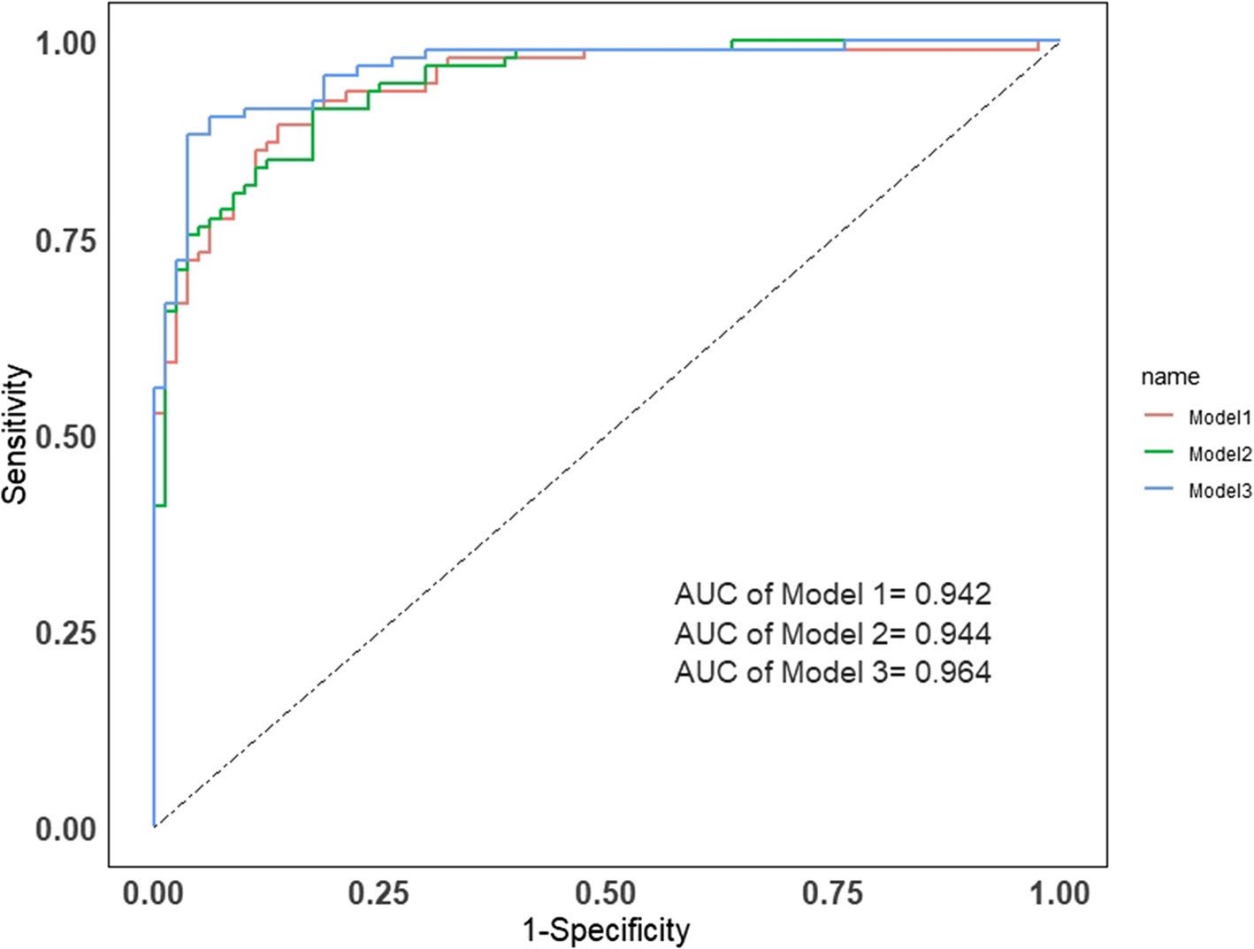
The ROC curves of 3 different diagnostic model. The areas under the ROC curves for the ability to differentiate TETs from thymic lymphomas for model 3(Age plus Symptoms plus SUVR) was 0.964. Model 1: Age plus SUVR; Model 2: Symptoms plus SUVR; Model 3: Age plus Symptoms plus SUVR

The diagnostic efficiencies of the PET/CT parameters were shown in Fig. 2 and Table 3 and the diagnostic models were shown in Fig. 3 and Table 4.

**Table 3:** Differential diagnostic efficiency of ^18^F-FDG PET/CT parameters between thymic epithelial tumors and thymic lymphomas

Parameters	Cut-off	AUC (95%CI)	Sensitivity (95%CI)	Specificity (95%CI)	PPV (95%CI)	NPV (95%CI)
**Lesion size (mm)**	74.5	0.775 (0.704–0.845)	0.763 (0.662–0.843)	0.725 (0.612–0.816)	0.763 (0.662–0.843)	0.725 (0.612–0.816)
**SUVmax**	10.5	0.845 (0.787–0.903)	0.742 (0.639–0.825)	0.850 (0.749–0.917)	0.852 (0.752–0.918)	0.739 (0.635–0.823)
**SUVmean**	6.2	0.835 (0.775–0.895)	0.688 (0.583–0.778)	0.863 (0.763–0.926)	0.853 (0.748–0.921)	0.704 (0.602–0.790)
**TLG**	626.7	0.822 (0.753–0.884)	0.688 (0.583–0.778)	0.850 (0.749–0.917)	0.842 (0.736–0.912)	0.701 (0.598–0.788)
**MTV**	113.9	0.730 (0.655–0.805)	0.624 (0.517–0.720)	0.775 (0.665–0.858)	0.763 (0.649–0.850)	0.639 (0.535–0.732)
**SUVR**	6.2	0.881 (0.831–0.932)	0.763 (0.662–0.843)	0.888 (0.792–0.944)	0.888 (0.792–0.944)	0.763 (0.662–0.843)

*CI* Confidence interval, *SUVmax* Max standard uptake value, *SUVmean* Mean standard uptake value, *MTV* Metabolic tumor volume, *TLG* Total lesion glycolysis, *AUC* Area under the curve, *PPV* Positive predictive value, *NPV* Negative predictive value, *SUVR* Standard uptake value ratio

**Table 4:** Differential diagnostic efficiency of different diagnostic models between thymic epithelial tumors and thymic lymphomas

Parameters	AUC (95%CI)	Sensitivity (95%CI)	Specificity (95%CI)	PPV (95%CI)	NPV (95%CI)
**Model 1**	0.942 (0.908–0.975)	0.892 (0.807–0.944)	0.863 (0.763–0.926)	0.883 (0.796–0.937)	0.873 (0.775–0.934)
**Model 2**	0.944 (0.914–0.975)	0.914 (0.832–0.959)	0.825 (0.720–0.898)	0.859 (0.771–0.917)	0.892 (0.793–0.949)
**Model 3**	0.964 (0.939–0.989)	0.882 (0.794–0.937)	0.963 (0.887–0.990)	0.965 (0.893–0.991)	0.875 (0.783–0.933)

Model 1: Age plus SUVR

Model 2: Symptoms plus SUVR

Model 3: Age plus Symptoms plus SUVR

*CI* Confidence interval, *AUC* Area under the curve, *PPV* Positive predictive value, *NPV* Negative predictive value

The addition of age and symptoms to SUVR allowed a significant reclassification with IDI = 0.271 (95% CI: 0.204–0.337, *P*<0.001) and categorical NRI = 0.338 (95% CI:0.186–0.490, *P*< 0.001), and model 3 allowed a significant reclassification with IDI and categorical NRI to model 2 (IDI = 0.095 [95% CI: 0.052–0.138, *P*<0.001]) and categorical NRI =0.163 [95% CI: 0.039–0.287, *P* = 0.001]). Model 2 allowed a significant reclassification with IDI (IDI = 0.093 [95% CI: 0.050–0.136, *P*<0.001]), but not with categorical NRI (Categorical NRI = 0.077 [95% CI: − 0.027-0.181, *P* = 0.148]). The DeLong test showed that the AUC of the model 3 was better than SUVR, model 1 and model 2 (*P*<0.001, *P* = 0.014, and 0.022, respectively). These results (Table 5) showed the benefits of statistical diagnostic with multiparametric combination in differential diagnosis of the TETs and thymic lymphomas.

**Table 5:** Comparison of the SUVR and different models to with DeLong’s test, IDI and NRI

Variable	DeLong’s test		IDI	95%CI	*P*	NRI	95%CI	*P*
	***Z***	***P***						
**Model 3 vs. SUVR**	3.87	<0.001	0.271	0.204–0.337	<0.001	0.338	0.186–0.490	<0.001
**Model 3 vs. Model 1**	2.45	0.014	0.093	0.050–0.136	<0.001	0.077	−0.027-0.181	0.148
**Model 3 vs. Model 2**	2.28	0.022	0.095	0.052–0.138	<0.001	0.163	0.039–0.287	0.010

Model 1: Age plus SUVR

Model 2: Symptoms plus SUVR

Model 3: Age plus Symptoms plus SUVR

*IDI* Integrated discrimination improvement, *NRI* Net reclassification improvement (categorical), *CI* Confidence interval

### Clinical application

**Fig. 4:**
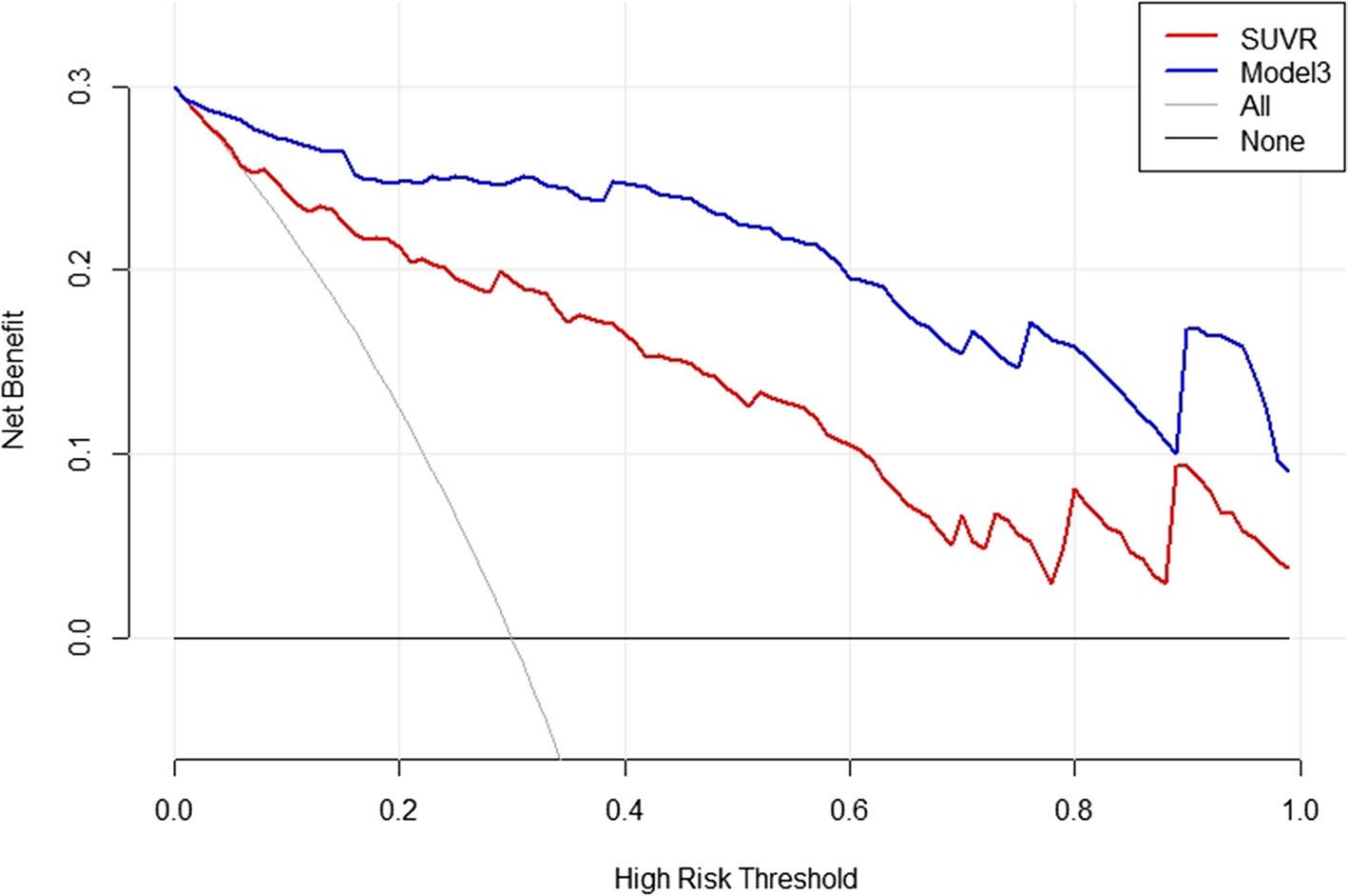
Decision curve analysis for combined diagnostic model 3 (age, symptoms and SUVR) and SUVR. The x-axis represents the threshold probability, and the y-axis represents the net benefit. The decision curve showed that regardless of the threshold probability of a doctor or a patient, using the combined diagnostic model in the current study to differential diagnosis of TETs and thymic lymphomas is more valuable than using SUVR alone

The decision curve analyses for the SUVR and the model 3 are presented in Fig. 4. Decision curve analyses showed that the model 3 had a higher overall net benefit than SUVR across all of the range of risk threshold.

## Discussion

In our study, we demonstrated that ^18^F-FDG PET metabolic parameters could differentiate TETs from thymic lymphomas, especially SUVR. Besides, we established a diagnostic model based on SUVR, age and clinical symptoms (including B symptom, myasthenia gravis, chest pain and respiratory symptoms), which could significantly improve the ability of differential diagnosis of TETs and thymic lymphomas.

TETs are rare tumors arising from thymic epithelial cells, and with the growth of age, the incidence rate of TETs is increasing, reaching its peak in the seventh decade of life [31]. Compared with TETs, the onset age of thymic lymphomas is younger, mostly between 10 and 39 years old [7]. Therefore, the age has a certain value in the differential diagnosis of TETs and thymic lymphomas. Patients with TETs are often accompanied by myasthenia gravis [32], while for patients with thymic lymphomas, B symptom (including weight loss > 10%, night sweats, body temperature > 38 °C) is the most typical clinical symptom [7, 33]. In our study, we found differences in age and clinical symptoms between patients with TETs and thymic lymphomas. Patients with thymus lymphomas were younger, and more prone to B symptoms and respiratory symptoms. Patients with TETs were more prone to myasthenia gravis and chest pain. Thymic lymphoma produces more respiratory symptoms, which may be due to rapid development, acute compression symptoms, and lesions larger than TETs, resulting in respiratory symptoms such as cough and dyspnea in more patients. For TETs, especially thymic carcinoma, the invasion of surrounding tissue and pleura may be more obvious [34], therefore the occurrence rate of chest pain was higher.

**Fig. 5:**
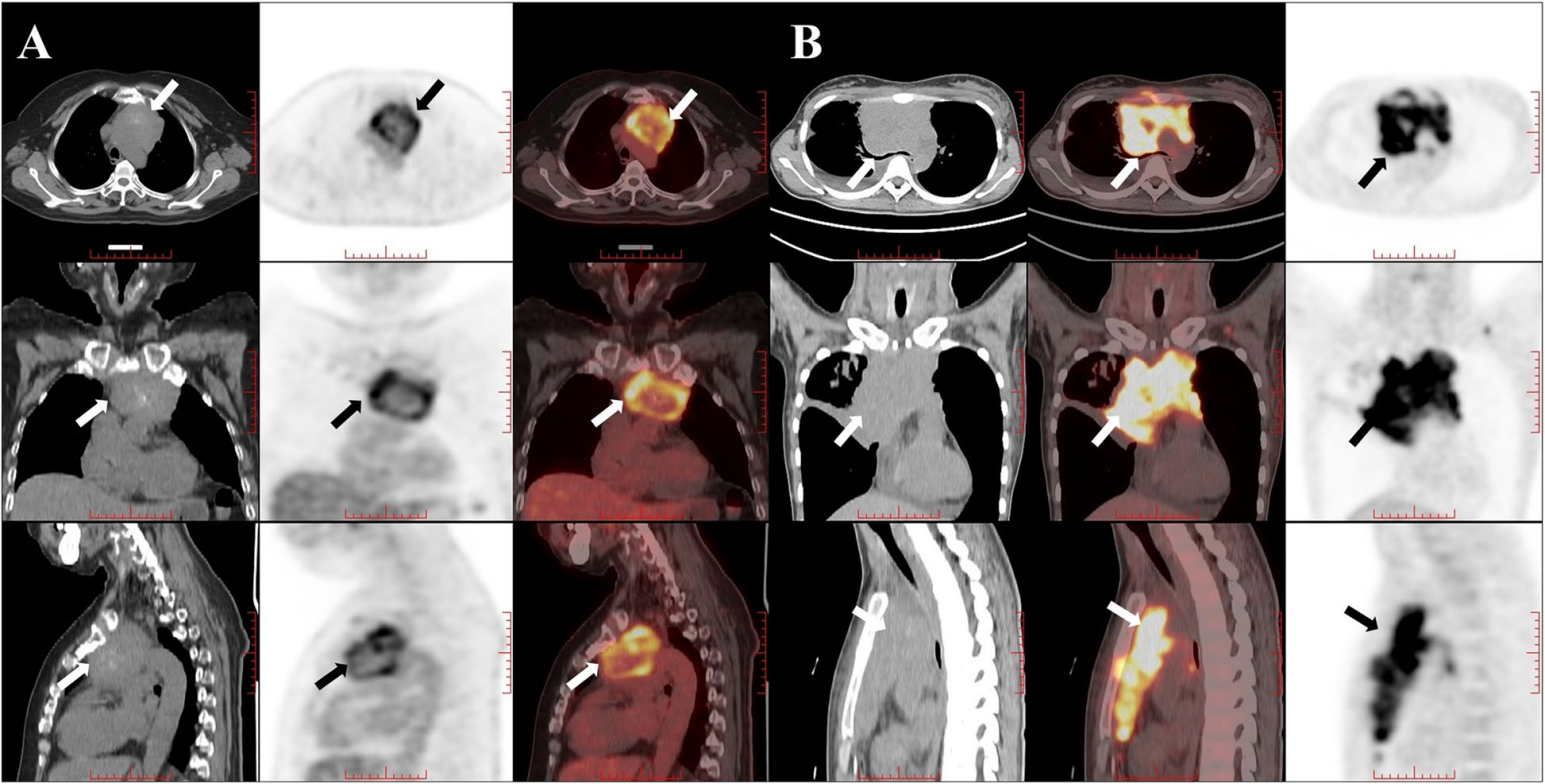
Image **A** in 59-year-old man with thymic squamous cell carcinoma (Masaoka Stage IIIB) in the anterior mediastinum (arrow). Enhanced CT showed that the boundary between the lesion and the blood vessel was not clear. Patient had chest and back pain. The lesion showed that SUVR was 2.49. Image **B** in 24-year-old woman with mediastinal diffuse large B-cell lymphoma (Ann Arbor Stage IVB) in the anterior mediastinum (arrow). Patient had respiratory symptoms (chest stuffiness) and B symptoms. The lesion showed that SUVR was 10.99

^18^F-FDG PET metabolic parameters have certain clinical value in the differential diagnosis of benign and malignant TETs [23, 35], staging [19, 36], prediction the grade of malignancy [37, 38], prediction of pathological response after induction therapy [39, 40], and prediction of prognosis [41, 42]. For thymic lymphomas, most studies about ^18^F-FDG PET mainly focus on staging [43], response evaluation [16, 44] and prognosis prediction [45–47]. SUVmax, as the most common semi-quantitative parameter of ^18^F-FDG PET, represents the highest glucose uptake in tumor or normal tissue, which is widely used in clinical practice. And volume-based variables, such as SUVmean, MTV and TLG, can reflect quantitatively the metabolic activity of the whole tumor. MTV and TLG are used to quantify the tumor burden of cancer patients, MTV represents the volume of the tumor with active FDG uptake, and TLG is calculated by multiplying the SUVmean of the total tumor by the metabolic tumor volume and represents both the tumor size and the extent of FDG uptake [48]. In our study, compared with TETs, thymic lymphomas had larger tumor size and higher FDG uptake (SUVmax), especially in large B-cell lymphoma and Hodgkin lymphoma, which are more invasive. Previous studies have also proved that the FDG uptake of thymic tumors with higher malignancy will be higher [13], and large B-cell lymphoma and Hodgkin lymphoma are two types of thymic lymphoma that are more common. Therefore, ^18^F-FDG PET metabolic parameters can be used in the differential diagnosis of TETs and thymic lymphomas. Previous studies have demonstrated that thymic cancer has higher FDG uptake than thymomas [22, 35, 49], SUVmax showed good diagnostic ability for differentiating high-risk thymoma/ thymic carcinoma from low-risk thymoma (AUC = 0.84, 95% CI: 0.76–0.92) and excellent ability for differentiating thymic carcinoma from low-risk thymoma/high-risk thymoma (AUC = 0.94, 95% CI: 0.90–0.98) [35]. According to our investigation, few previous studies investigated the differential diagnosis of TETs and thymic lymphoma by using ^18^F-FDG PET metabolic parameters. Zhu et al. applied metabolic parameters in differential diagnosis of 71 patients with primary mediastinal lymphomas (PMLs) and 65 patients with TETs [23]. Patients with PMLs had higher SUVmean, SUVmax, TLG, and MTV values than patients with TETs. ROC analysis indicated that the SUVmax (AUC = 0.767, sensitivity = 70.4%, specificity = 70.8%) and SUVmean (AUC = 0.764, sensitivity = 76.1%, specificity = 69.3%) performed similarly in differentiating patients with PMLs from TETs, and both values were better than the MTV and TLG values. This result was similar to our results, our results indicated that SUVR (AUC = 0.881), SUVmax (AUC = 0.845) and SUVmean (AUC = 0.835) had considerable discrimination ability, and were better than TLG (AUC = 0.822) and MTV (AUC = 0.730). This suggested that metabolic parameters, especially SUVR, could be of certain value in the differentiation of TETs and thymic lymphomas (Fig. 5).

Due to the differences of age and symptoms between TETs and thymic lymphoma, as well as the good diagnostic ability of metabolic parameters, we combined age, symptoms and SUVR to form a diagnostic model. Previous study combining age and SUVmean demonstrated the ability in the differential diagnosis of TETs and thymic lymphomas, with high sensitivity (83.1%) and specificity (88.7%) [23]. In our study, through NRI, IDI and Delong test, the application of model 3 could significantly improve the differential diagnostic ability compared to SUVR. And model 3 also shows better diagnostic ability than model 1 and model 2. Decision curve analyses suggested that differential diagnosis of TETs and thymic lymphomas using the diagnostic model in the current study was more valuable than using SUVR alone, regardless of the physician’s or patient’s threshold probability. Composition model based on SUVR, age and clinical symptoms could achieve very excellent diagnostic efficiency (AUC = 0.964, sensitivity = 0.882, specificity = 0.963), and provided additional information in staging, and reliable evidence for patients’ treatment choice.

This study had some limitations. First, although this study included the largest number of cases compared with the relevant available studies, this was still a retrospective cohort study. And most patients (one hundred and twenty-eight thymic lesions) did not undergo surgery, which was proved by puncture biopsy. These may lead to bias of the statistics and diagnostic model. Second, the SUV is influenced by many factors [50], this may lead to a certain non-repeatability of the model constructed with metabolic parameters in diagnosis. Because this is a two-center study, the measurement of metabolic parameters may be different due to machine parameters and ^18^F-FDG injection dose. Therefore, we corrected this issue through SUVR to minimize the result bias. In the future, we will continue to add PET image radiomics related parameters such as texture parameters to further improve the stability and repeatability of the model.

## Conclusion

In general, the multiparameter diagnostic model composed of age, clinical symptoms and ^18^F-FDG PET metabolic parameters has excellent diagnostic efficacy in the differential diagnosis of TETs and thymic lymphomas. Our results provided a more accurate and reliable evaluation for the differential diagnosis of preoperative thymic tumors, which can avoid more patients receiving unnecessary treatment and surgery.

## Supplementary Information

**Additional file 1: Table S1.** The ^18^F-FDG PET/CT parameters of different thymic tumors.

## Data Availability

The datasets used and/or analysed during the current study are available from the corresponding author on reasonable request.

## Abbreviations

^18^FDG PET: ^18^F-fluorodeoxyglucose positron emission tomography
TETs: Thymic epithelial tumors
SUVmax: Maximum standard uptake value
SUVmean: Mean standard uptake value
TLG: Total lesion glycolysis
MTV: Metabolic tumor volume
SUVR: Tumor-to-normal liver standard uptake value ratio
IDI: Integrated discriminatory improvement
NRI: Net reclassification improvement
DCA: Decision curve analysis
PPV: Positive predictive value
NPV: Negative predictive value
GCTs: Germ cell tumors
NETs: Thymic neuroendocrine tumors
PMBCL: Primary mediastinal large B-cell lymphoma
NSHL: Nodular sclerosis Hodgkin lymphoma
T-LBL: T-cell lymphoblastic lymphoma
WHO: The World Health Organization
AC: Attenuation correction
VOI: Volume of interest
