# Supplementary material for: Multiparameter diagnostic model based on 18F-FDG PET and clinical characteristics can differentiate thymic epithelial tumors from thymic lymphomas
Source: BMC Cancer. 2022 Aug 16;22:895. doi: 10.1186/s12885-022-09988-1 (PMC9382789; doi:10.1186/s12885-022-09988-1)
Supplement: Supplementary file 1 — Additional file 1: Table S1. The 18F-FDG PET/CT parameters of different thymic tumors. [file 12885_2022_9988_MOESM1_ESM.docx]

**Table-S1.** The ^18^F-FDG PET/CT parameters of different thymic tumors

|  | LRT  (n=11) | HRT  (n=17) | TC  (n=44) | NETs  (n=8) | Large B-cell lymphoma (n=37) | Hodgkin lymphoma (n=31) | T lymphoblastic lymphoma (n=23) | MALT  lymphoma (n=1) | ALCL lymphoma (n=1) |  |
| --- | --- | --- | --- | --- | --- | --- | --- | --- | --- | --- |
| Lesion size (mm) | | 41.0±14.6 | 59.9±26.9 | 72.4±32.2 | 59.3±43.9 | 104.9±35.8 | 82.4±31.3 | 115.6±43.0 | 69 | 124 |
| SUVmax | | 3.5±1.7 | 5.1±2.1 | 9.0±4.7 | 7.1±3.0 | 19.1±7.3 | 14.0±5.0 | 11.8±8.4 | 2.9 | 24.8 |
| SUVmean | | 2.1±1.0 | 3.1±1.4 | 5.0±2.8 | 4.1±1.6 | 10.9±4.7 | 8.2±3.1 | 6.3±4.6 | 1.7 | 12.9 |
| TLG | | 103.7±108.7 | 262.1±297.2 | 486.0±577.4 | 276.0±355.2 | 2761.1±2076.7 | 772.1±712.5 | 2201.3±2493.6 | 224.4 | 2324.3 |
| MTV | | 50.0±36.6 | 73.1±71.7 | 116.2±154.0 | 59.5±78.4 | 262.4±195.3 | 91.9±75.3 | 364.0±401.3 | 129.0 | 180.7 |
| SUVR | | 1.7±0.8 | 2.5±1.2 | 4.7±2.6 | 3.4±1.7 | 12.5±5.0 | 9.0±3.8 | 9.3±9.5 | 1.6 | 15.6 |

LRT: Low-risk thymoma; HRT: High-risk thymoma; TC: Thymic carcinoma; NETs: Thymic neuroendocrine tumors; MALT lymphoma: Extranodal marginal zone lymphoma of mucosa associated lymphoid tissue; ALCL lymphoma: Anaplastic large cell lymphoma

We compared the ^18^F-FDG PET/CT parameters of LRT, HRT, TC, NETs, large B-cell lymphama, Hodgkin lymphama and T lymphoblastic lymphoma (according to whether the variance was homogeneous, ANOVA or Kruskal-Wallis H test was applied respectively), and the results showed that there were significant differences in all ^18^F-FDG PET/CT parameters among the groups (*P*＜0.001).
